# Towards combating antibiotic resistance by exploring the quantitative structure-activity relationship of NDM-1 inhibitors

**DOI:** 10.17179/excli2022-5380

**Published:** 2022-11-16

**Authors:** Tianshi Yu, Aijaz Ahmad Malik, Nuttapat Anuwongcharoen, Warawan Eiamphungporn, Chanin Nantasenamat, Theeraphon Piacham

**Affiliations:** 1 Center of Data Mining and Biomedical Informatics, Faculty of Medical Technology, Mahidol University, Bangkok 10700, Thailand; 2 Department of Clinical Microbiology and Applied Technology, Faculty of Medical Technology, Mahidol University, Bangkok 10700, Thailand; 3 Center of Excellence in Computational Molecular Biology, Faculty of Medicine, Chulalongkorn University, Bangkok, Thailand; 4 Streamlit Open Source, Snowflake Inc., USA

**Keywords:** antibiotic resistance, beta-lactamase, NDM-1, QSAR, drug discovery, data science

## Abstract

The emergence of New Delhi metallo-beta-lactamase-1 (NDM-1) has conferred enteric bacteria resistance to almost all beta-lactam antibiotics. Its capability of horizontal transfer through plasmids, amongst humans, animal reservoirs and the environment, has added up to the totality of antimicrobial resistance control, animal husbandry and food safety. Thus far, there have been no effective drugs for neutralizing NDM-1. This study explores the structure-activity relationship of NDM-1 inhibitors. IC_50_ values of NDM-1 inhibitors were compiled from both the ChEMBL database and literature. After curation, a final set of 686 inhibitors were used for machine learning model building using the random forest algorithm against 12 sets of molecular fingerprints. Benchmark results indicated that the KlekotaRothCount fingerprint provided the best overall performance with an accuracy of 0.978 and 0.778 for the training and testing set, respectively. Model interpretation revealed that nitrogen-containing features (KRFPC 4080, KRFPC 3882, KRFPC 677, KRFPC 3608, KRFPC 3750, KRFPC 4287 and KRFPC 3943), sulfur-containing substructures (KRFPC 2855 and KRFPC 4843), aromatic features (KRFPC 1566, KRFPC 1564, KRFPC 1642, KRFPC 3608, KRFPC 4287 and KRFPC 3943), carbonyl features (KRFPC 1193 and KRFPC 3025), aliphatic features (KRFPC 2975, KRFPC 297, KRFPC 3224 and KRFPC 669) are features contributing to NDM-1 inhibitory activity. It is anticipated that findings from this study would help facilitate the drug discovery of NDM-1 inhibitors by providing guidelines for further lead optimization.

## Introduction

Antibiotic resistance is defined as bacteria having resistance to the effects of antibiotic agents. It has already been listed in the top 10 global public health burdens by the WHO. Every year in the US, there are more than 2.8 million cases of infection by antibiotic-resistant bacteria, and mortality is more than 35000. From amongst all antibiotics, almost 70% of pathogenic bacteria have developed resistance to at least 1 antibiotic (Bush and Bradford, 2016[6]).

Beta-lactamases are enzymes secreted by bacteria to hydrolyze the most widely used antibiotics: beta-lactam antibiotics, rendering them useless and posing a major threat to antibiotic resistance control. There are two categories of beta-lactamases: serine-active-site and zinc-ion-active-site beta-lactamases, according to the Ambler classification system. Beta-lactamases with serine active sites belong to classes A, C and D and those with zinc-ion active sites belong to class B (Bahr et al., 2021[1]). Because the active sites of class B beta-lactamases are coordinated by metal ions, they are also called metallo-beta-lactamases (MBL). Amongst MBLs, New Delhi Metallo-β-lactamase-1 (NDM-1) was first discovered in *K. pneumoniae* and *E. coli* of a Swedish traveller back home from India in the year 2008. *K. pneumoniae* and *E. coli* harboring NDM-1 have spread widely in the Indian subcontinent, China, Southeast Asian countries, and the Middle East. Until now there are a total of 41 genetic variants of NDM-1, ranging from NDM-1 to NDM-41. In comparison with other significant metallo-beta-lactamases such as Imipenemase (IMP-1) and Verona integron-encoded (VIM-2), NDM-1 is mainly harbored by Enterobacteriaceae species (especially *K. pneumoniae* and *E. coli*), while IMP-1, VIM-2 are harbored by *P. aeruginosa* (Naas et al., 2017[55]; Eiamphungporn et al., 2018[17]). Anchored to the outer membrane of gram-negative bacteria as a lipoprotein, NDM-1 possesses higher tolerance of zinc ion depletion than other MBL members as water-soluble periplasmic enzymes. In addition, NDM-1 has highly efficient hydrolytic capabilities against a wide spectrum of beta-lactams, including carbapenems (González et al., 2016[24]). 

Clinical bacterial strains expressing NDM-1 are currently susceptible only to certain antibiotics of last resort: colistin, tigecycline, and fosfomycin (Tooke et al., 2019[80]). All those antibiotics of last resort demonstrate significant side effects, which limit their large-scale application. In addition, NDM-1 and its genetic variants can be transmitted by plasmids, which can be facilitated by accelerated globalization and international travellers. Last but not least, plasmids harboring NDM-1 genes are reported to be identified amongst food animals and the environment (Wang et al., 2012[85]; Szmolka and Nagy, 2013[77]; Islam et al., 2017[33]; Parvez and Khan, 2018[60]). The isolation of blaNDM-1 genes in food animals indicates the zoonotic transmission capability and implicates significant food safety issues. While the environmental spread of blaNDM-1, especially through water, poses more pressure on the environmental containment of antimicrobial resistance. All the above aspects of NDM-1 make infection control rather time and resource-consuming.

Until now, although there is noticeable progress on drug discovery for NDM-1 inhibiting molecules, there are no effective inhibitors against NDM-1 that can be applied in clinical practice. Some conventional beta-lactamase inhibitors, such as clavulanic acid, sulbactam, and tazobactam cannot inhibit NDM-1. There are a number of inhibitors *in vitro*, for example, L-captopril, Aspergillomarasmine A, cyclic boronates, etc (Li et al., 2014[45]; Hecker et al., 2020[29], Tehrani et al., 2020[79]). Drug discovery for NDM-1 inhibitors faces a series of difficulties, due to the unique structural characteristics of NDM-1, including flexibility of the binding pocket, adaptability of the active site loop, the open shallow cavity of the active site, water reorganization upon ligand binding, relative lacking of knowledge of whole catalysis process and potential off-target effects to physiological metalloenzymes, etc. All the above make the drug discovery of NDM-1 inhibitors highly challenging and uncertain (Behzadi et al., 2020[2]).

Quantitative structure-activity relationship (QSAR) is a ligand-based drug discovery approach that harnesses mathematical models for correlating physicochemical property information of molecules with their biological activities. The essence of QSAR is based on two principles: (i) structure dictates activity and (ii) molecules with similar structures demonstrate similar bioactivities (Tropsha, 2010[81]). As a methodology in chemistry and drug discovery, QSAR/QSPR has gone through a remarkable transformation since its dawn 60 years ago. As early as the time of Corwin Hansch, QSAR/QSPR modeling processes were performed on a small number of molecules with few molecular descriptors employing multilinear regression. For now, thanks to the development of information technology and artificial intelligence, QSAR/QSPR has evolved to the application of a large dataset, equipped with sophisticated molecular descriptors, advanced machine learning algorithms, and various validation techniques. QSAR/QSPR modeling techniques are now widely used in chemistry, drug discovery, material science, and environmental protection. And they are not for bioactivities only, but also chemical properties prediction, for example, melting point, biodegradation rate, ecotoxicity, blood-brain-barrier penetration, etc (Nantasenamat and Prachayasittikul, 2015[57]; Nantasenamat et al., 2015[58]; Nantasenamat, 2020[56]). Previously, there are some studies on the structure-activity relationship of specific series of NDM-1 inhibitors, for example, Aspergillomarasmine A derivatives, rhodanines and derived enethiol inhibitors, azetidinimines, dipicolinic acid derivatives (Chen et al., 2017[10]; Zhang et al., 2017[94], 2018[93]; Romero et al., 2021[63]). However, until now, there is no structure-activity relationship study on the comprehensive library of NDM-1 inhibitors. 

The OECD has established principles for QSAR modeling consisting of five rules: defined endpoint, unambiguous algorithms, defined applicability domain, model validation, and mechanistic interpretation. The rules involve various steps of QSAR modeling: data compilation, data splitting, machine learning process, evaluation of the robustness and predictability of the model, and mechanistic interpretation of feature importance (Fjodorova et al., 2008[20]; Tropsha, 2010[81]; Piir et al., 2018[61]). In this study, a QSAR classification model was built according to the OECD criteria using the random forest algorithm for investigating the quantitative structure-activity relationship for NDM-1 inhibitors. After hyperparameter optimization, the model was interpreted by selecting important features for exploring their contributions and mechanisms in NDM-1 inhibiting activities.

## Materials and Methods

**Figure 1:**
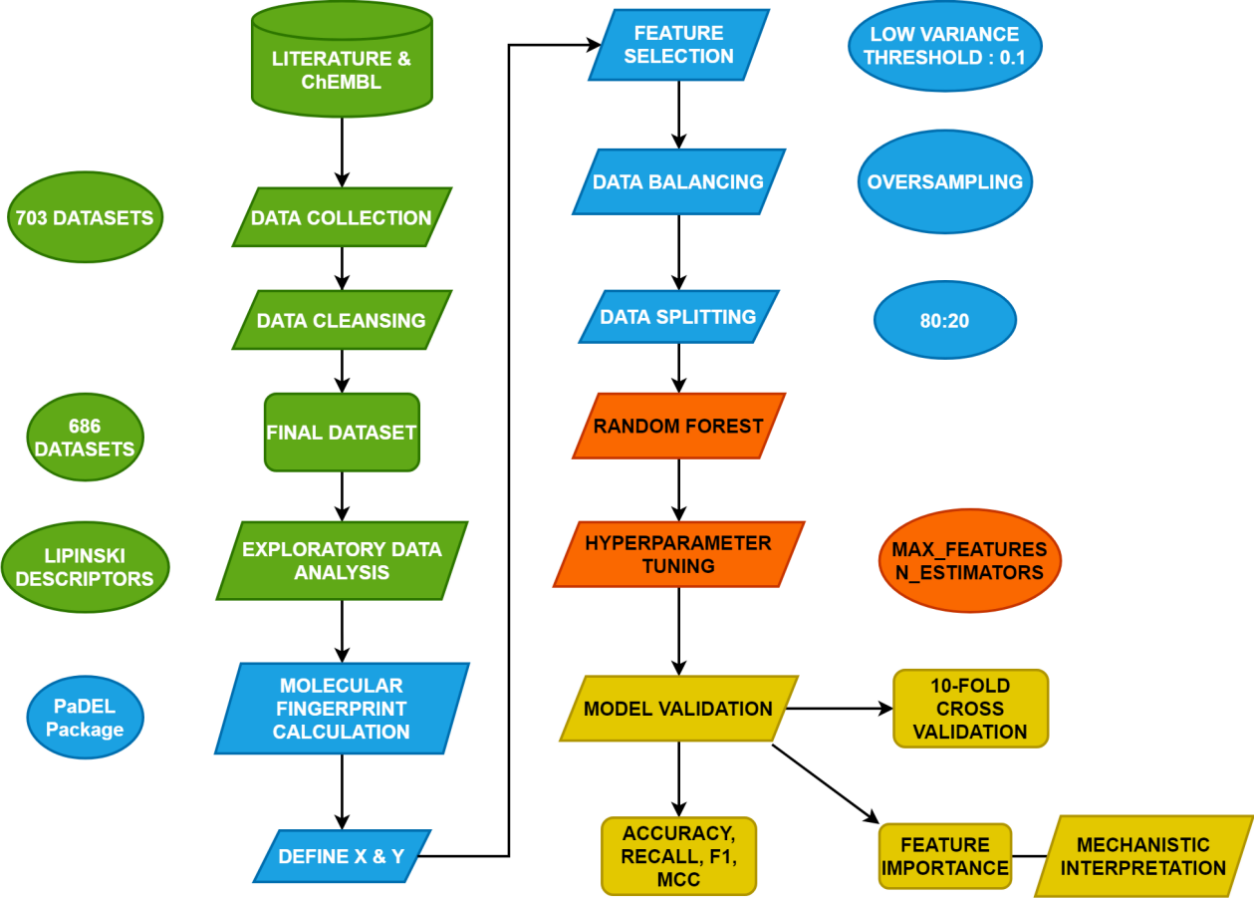
Schematic diagram of the conceptual framework of this study. Different colors symbolize various steps of the study. Green means data collection and data cleansing, blue means descriptor calculation, red means QSAR modeling and orange means validation and mechanistic interpretation.

This is a computational study utilizing QSAR modeling for investigating the quantitative structure-activity relationship for NDM-1 inhibitors. QSAR modeling aims to establish a relationship between the intrinsic information of molecules and their endpoint bioactivity values/classes. The design of this study is summarized in schematic diagram in Figure 1(Fig. 1).

### Data compilation

All biological activity data sets are isolated from the ChEMBL database and a compilation of primary literature. The complete list of origins of data sets are shown in Supplementary Table 1. IC_50_ values are selected exclusively in this study for further investigation. As a result, 703 NDM-1 inhibitor activities have been obtained. After data cleansing which involves removal of redundant data, removal of unqualified data, imputation of missing or unqualified data, there are 686 non-redundant molecules left with available IC_50_ values as the final data sets for modeling. And above all, for the sake of more straightforward visibility of the bioactivities and interpretability of the values, needs to transform IC_50_ to pIC_50_ values. pIC_50_ is the negative logarithmic value of IC_50_, and the transformation of value can make the data logarithmic, demonstrating the distribution, diversity, and tendency more visible. Particularly, IC_50_ ≤ 1μM (pIC_50_ ≤ 6) were considered as active, while IC_50_ > 10 μM (pIC_50_ < 5) inactive. Those IC_50_ between them are considered intermediate. Shown in Table 1(Tab. 1) is the summary of counts of all bioactivity classes.

### Molecular fingerprint descriptor calculation

Molecular fingerprints are the representations of a complex form of molecular descriptors. In this study, the PaDEL package was used for calculating 12 sets of molecular fingerprints (Yap, 2011[90]). Details are shown in Table 2(Tab. 2). 

Klekota-Roth fingerprints is a set of 4860 molecular substructures proposed by Justin Klekota and Fredrick Roth. These substructures originated from chemical libraries and are explored for their privileges in bioactivities. PubChem fingerprints encode molecular fragments information with 881 binary digits and can be accessed from PubChem. Substructure fingerprint set consists of 307 chemical functional groups by SMARTS patterns. The molecular fingerprint means the presence or absence of any particular fingerprint, and the word count means the number of these particular fingerprints (Yap, 2011[90]). Considering the interpretability for biologists, only KlekotaRoth, KlekotaRothCount, PubChem, Substructure and SubstructureCount fingerprint sets are used for further modeling.

As each set of fingerprints contains tens even hundreds of structural datasets, they can add up to total complexity and give the risk of bias in the model. Therefore, feature selection is necessitated by removing low variance features. By default, all zero variance features should be removed, as zero-variance features have constant value and all instances share the same constant on this feature. The threshold of variance is set to 0.1, which means that features with variance lower than 0.1 are to be removed, without affecting the overall performance of the model, as well as reducing the time and computational resources (Nantasenamat, 2020[56]). 

### Data balancing and data splitting

**Figure 2:**
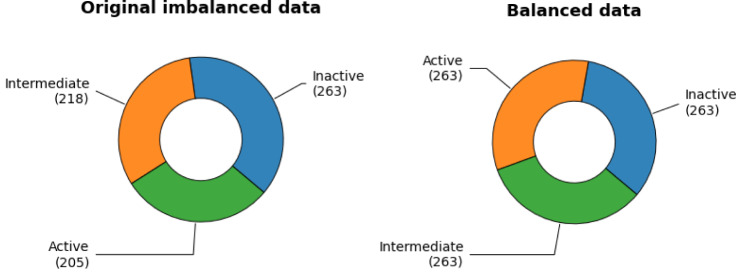
Schematic comparison of original and balanced dataset

Data balancing is a critical step in classification modeling. Data imbalance occurs when the classes of the dataset are distributed unequally. For classification models, data imbalance is too common to be avoided. Generally, the influence of a mild degree of data imbalance can be ignored, while the influence of significant data imbalance can lead to the unreliability of accuracy. To handle data imbalance, in this study, random oversampling technique is used. Random oversampling by essence is the random duplication of the minority class amongst datasets. Shown in Figure 2(Fig. 2) is the comparison between imbalanced data and balanced data. Data splitting is a validation procedure based on the division of the input data set into a training set and a test set (Nantasenamat et al., 2015[58]). In this study, the training set and the testing set will be in the ratio of 80:20. Afterwards, 10-fold cross-validation was performed, as the internal validation procedure, so as to make the most use of the data for ensuring the robustness and reliability of the model.

### QSAR modeling and hyperparameter tuning

As a multivariate analysis utilizing machine learning automatic modeling to correlate the independent variables (molecular fingerprint information) with dependent variables (bioactivities), in this study, the QSAR modeling is done by employing a random forest algorithm, through the Jupyter notebook, based on Python scripts. In order to ensure the reproducibility of the model, all the random states during the programming are set to 42 by default. 

Hyperparameters of the random forest algorithm can be tuned in order to optimize the model performance. Unlike parameters of a model that can be learned during the training process, hyperparameters are set up before the training begins. In this study, two significant hyperparameters from the random forest algorithm, i.e., the number of decision trees in the forest (n_estimators) and the number of features considered by each tree when splitting a node (max_features) are selected for tuning. The reason as to why they are selected are described as follows. The greater the number of decision trees in the random forest, the better performances they can bring about. However, the increasing number of decision trees can consume more computational resources and slow down the modeling process, without optimizing the model performance significantly. Meanwhile, the number of maximal features by each tree when splitting can impact the model performance to various degrees depending on the size and characters of the dataset. In the next step, GridSearchCV from the scikit-learn Python library is used to evaluate the model performance in a grid-wise manner by exhaustively combining all hyperparameters in the selected range. In this study, the n_estimators hyperparameter is set to 0-700, using 20 as step size. As for the max_features hyperparameter, the employed value range is set to 1-5. The combination of hyperparameters that provide the best performance is selected for modeling. 

### Model validation

In order to evaluate the robustness and generalization of the model, validation is performed as an indispensable step of modeling. The measure of validation consists of internal validation and external validation. The internal validation is the validation within the training set, mostly by cross validation. Meanwhile, the external validation is the validation of the holistic model (Shoombuatong et al., 2018[71]). 

For classification models, there are a series of parameters to validate the model. In this study, accuracy (ac), recall (re), f1 score (f1) and MCC (Matthew's correlation coefficient) are used for validation. For each set of descriptor/fingerprint, the model will be validated for their performances both in the training set and testing set. The outstanding model will be extracted and further validated through cross validation.


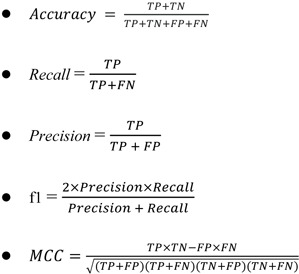


### Mechanistic interpretation of feature importance 

Feature importance refers to assigning a score to the input variables based on how important they are at contributing to the target variable. Random forest algorithm has built-in feature importance of Gini importance (or mean decrease impurity), which is computed from the random forest structure. As random forest algorithm consists of a multitude of decision trees, and each tree can be seen as a set of internal nodes and leaves, the internal nodes determine how to make decisions by dividing datasets into 2 separate subsets. Features for the internal nodes are selected by Gini impurity or information gain in classification model, or variance reduction in regression model. The random forest built-in feature importance is computed during the process of modeling, so that it can save lots of time and computing resources. 

### Reproducible research

Reproducibility is defined as the 'closeness of the agreement between the results of measurements of the same measure and carried out under changed conditions of measurement'. Reproducibility of the experiment, whether *in vitro* or *in silico*, is a major concern in science and technology as it is closely related to extensibility of knowledge and reproducibility of outputs (Schaduangrat et al., 2020[66]). As this is a computational study, to maintain reproducibility of the model, all the data sets, source codes are uploaded to GitHub repository, and all random seeds are set at 42. All the above information can be accessed at https://github.com/georgeyuricadd/ic50-dataset.

## Results and Discussions

The emergence of NDM-1 has conferred pathogenic bacteria almost full spectrum resistance to beta-lactam antibiotics. However, until now there are no available NDM-1 inhibiting drugs that are applied in practice. Due to the gravity of the global antimicrobial resistance burden, there is an urgent need to develop new drugs to inhibit NDM-1. This study is a QSAR study built according to the OECD criteria using the random forest algorithm for investigating the quantitative structure-activity relationship for NDM-1 inhibitors. After using 12 sets of molecular fingerprints from the PaDEL package for classification model, the KlekotaRothCount fingerprint set stands out with the best model performance. After modeling, hyperparameter tuning and feature importance ranking, top 20 ranked fingerprints, including nitrogen-containing features (KRFPC 4080, KRFPC 3882, KRFPC677, KRFPC3608, KRFPC 3750, KRFPC4287 and KRFPC3943), sulfur-containing substructures (KRFPC 2855 and KRFPC 4843), aromatic features (KRFPC 1566, KRFPC 1564, KRFPC 1642, KRFPC 3608, KRFPC 4287 and KRFPC 3943), carbonyl features (KRFPC 1193 and KRFPC 3025), aliphatic features (KRFPC 2975, KRFPC 297, KRFPC 3224 and KRFPC 669) are used for mechanistic interpretation of feature importance.

As a reproducible QSAR model to explore the structure-activity relationship of NDM-1 inhibitors, this study utilizes a comprehensive dataset source, interpretable learning algorithm and measurable evaluation metrics for building the model. In addition, after mechanistic interpretation of feature importance, the discoveries from the important features largely provide suggestions and directions for further NDM-1 drug discovery. From a larger point of view, the results and discoveries of the study facilitate combat against antimicrobial resistance.

### Exploratory data analysis and visualization 

**Figure 3:**
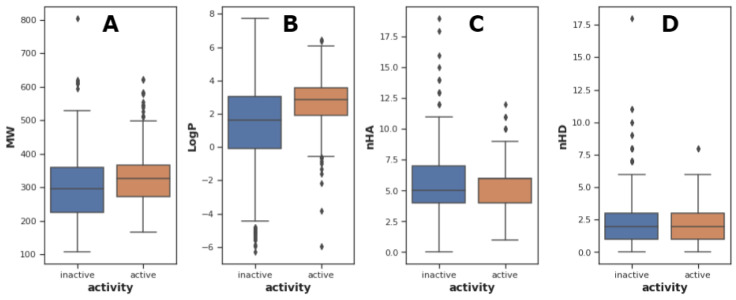
Distribution of Lipinski's descriptors by bioactivities in which all the Lipinski descriptors including A) molecular weight (MW), B) lipophilicity (LogP), C) number of hydrogen bond acceptor atom (nHA) and D) number of hydrogen bond donor atom (nHD) are calculated and compared between inactive and active classes.

An exploratory data analysis is done to visualize the distribution, diversity, and patterns of the NDM-1 inhibitors. Amongst the 686 inhibitors, there are 263 inactive molecules, 205 active molecules, and 218 intermediate molecules. The molecules are evaluated and compared with Lipinski descriptors as shown in Figure 3(Fig. 3).

Lipinski's rule-of-five is a statistical principle to evaluate the drug-likeness properties of orally available drugs by Christopher Lipinski (Lipinski et al., 2001[47]). It consists of molecular weight no more than 500 kDa; octanol-water partition coefficient no more than five; hydrogen bond acceptors no more than ten; hydrogen bond donors no more than five. As a statistical principle, the set of rules is not an absolute principle to define or exclude a molecule as a drug. A molecule that meets all criteria of Lipinski's rule doesn't make it a drug, and vice versa, a molecule that violates 1 or 2 rules of Lipinski's rule doesn't exclude its possibility to be a drug. For example, some prominent drugs such as atorvastatin and montelukast, both violate more than one of the Lipinski rules (Bickerton et al., 2012[3]). According to medicinal chemistry, all drugs play pharmaceutical effects via 3 aspects: molecular shape and size; electronic effects, and solubility profiles. Hereby, molecular weight largely determines the molecular size and shape; nHA and nHD are the electronic effects, and LogP corresponds to solubility profiles. In exploratory data analysis, Lipinski's rule of five properties is evaluated and visualized with regard to the bioactivities of the molecules. From Figure 3(Fig. 3), it is clear that the distribution of most molecules in the Lipinski descriptor chemical space satisfies Lipinski criteria of drug likeness. All the descriptors are validated to abide by nonparametric distributions.

When performing Mann-Whitney U test between the two classes, molecular weight gets *p*-value=6.99e-05, LogP gets *p*-value=5.90e-10 and nHD gets *p*-value=0.058, while the *p*-value for nHA is 0.283. The *p*-values calculated from Mann-Whitney U test indicate the statistical significance of differences between classes. As seen from Table 3(Tab. 3), the overall molecular weight and LogP of molecules in the active class are higher than those in the inactive class. While, the number of hydrogen bond acceptors and donors amongst the two classes are not significantly different.

### Classification model and hyperparameter tuning

Classification modeling aims to establish the relationship between molecular fingerprints and bioactivity classes. All 12 sets of fingerprints are used to build the model. Random forest algorithm is set by default to include estimators=500, max_features=3 with random state 42. In Table 4(Tab. 4), the performance metrics with default hyperparameters is listed. From the table, KlekotaRothCount fingerprint provides the best overall accuracy, recall, F1 score and MCC values. And due to its interpretability for biologists and pharmacists, it is used for the classification model and feature importance.

**Figure 4:**
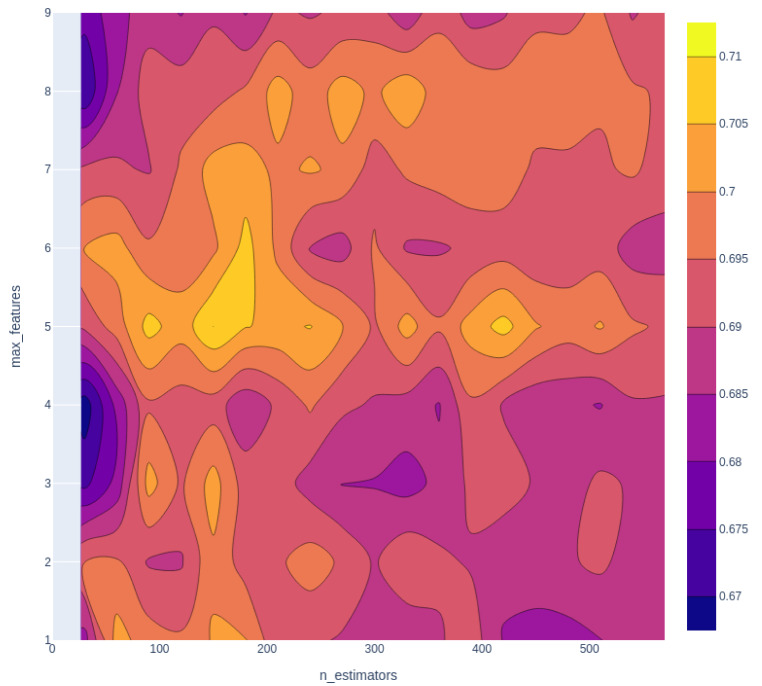
Hyperparameter tuning for random forest classifier. The lighter the color, the better the combination of the hyperparameters, from the figure, hyperparameter n_estimators =150, max_features=5 generates the best performance.

**Figure 5:**
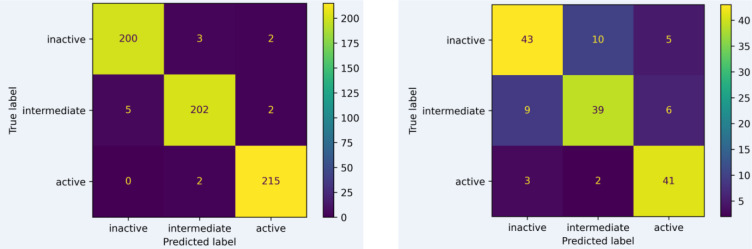
Confusion matrix of experimental versus predicted bioactivity classes of model built using KlekotaRothCount fingerprints as evaluated on training (A) and test (B) sets after hyperparameter tuning

After selecting KlekotaRothCount fingerprint as the one for further modeling, hyperparameters are tuned to optimize the model performance. The results for hyperparameter tuning are demonstrated in Figure 4(Fig. 4). The optimal hyperparameter is n_estimators = 150, and max_features = 5. After that, the confusion matrix of its performance is shown in Figure 5(Fig. 5). 

Then the optimized parameters are applied to the KlekotaRothCount fingerprint, and the model performance gets improved as shown in Table 5(Tab. 5). 

### Feature importance

**Figure 6:**
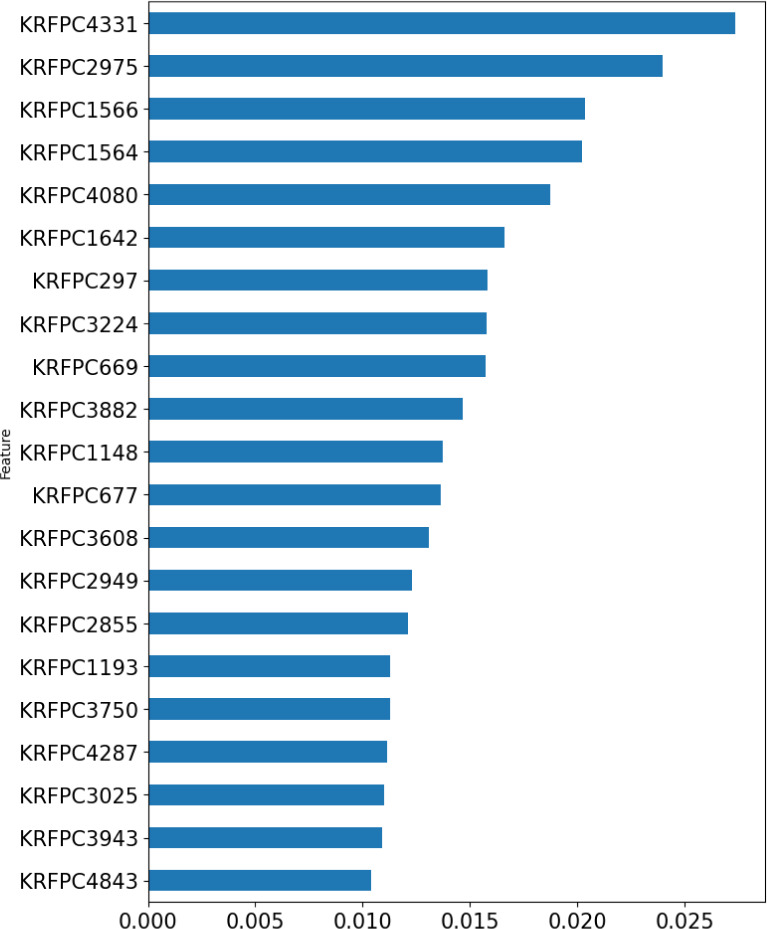
Bar plot of feature importance of the classification model. The top 20 KlekotaRothCount fingerprints are demonstrated in the horizontal bar chart.

Shown in Figure 6(Fig. 6) is the intrinsic feature importance ranking of the random forest algorithm. Top 20 ranked features are selected for mechanistic interpretation.

As can be seen from the above feature importance plot, the 20 top-ranked features are listed and described in Table 6(Tab. 6). Based on the nature of these KlekotaRothCount fingerprints, which can be categorized as nitrogen-containing features, sulfur-containing features, aromatic features, carbonyl group features, and aliphatic features. 

### Mechanistic interpretation of feature importance 

#### Mechanistic interpretation of selected features

NDM-1, as a metallo-beta-lactamase, targets amide bonds via nucleophile attack on the carboxyl carbons to hydrolize the substrate antibiotics. NDM-1 has the typical 4-layer sandwich conformation, with 2 β-sheets in the middle, surrounded by 4 α-helixes, and two zinc ions in the active sites (Linciano et al., 2019[46]). Almost all of the zinc ion coordination residues amongst metallo-beta-lactamases are highly conserved. In NDM-1, zinc1 is coordinated by H116, H118, H196, forming a tetrahedral sphere along with a water molecule, and the zinc2 is coordinated by D120, C221, H263, forming trigonal pyramidal sphere along with two water molecules (Linciano et al., 2019[46]). Due to the key roles of zinc ions and the highly conserved zinc-coordinating residues, they have become the primary targets of concurrent NDM-1 inhibitors (Wang et al., 2021[83]). 

Inhibitors targeting the active site of NDM-1 work by binding to the zinc ions to form ternary complexes to competitively inhibit the hydrolysis of substrate antibiotics, or by stripping zinc ions from the enzyme so as to prevent the enzyme from launching nucleophile attack on the substrate antibiotics. In this study, NGL viewer, Poseview and LigPlot, are used to visualize and analyze the mechanism of action of NDM-1 inhibitors and confirm the role of selected important features in NDM-1 inhibition (Stierand et al., 2006[74]; Laskowski and Swindells, 2011[41]; Fährrolfes et al., 2017[18]; Rose et al., 2018[64]). 

#### Nitrogen-containing features

**Figure 7:**
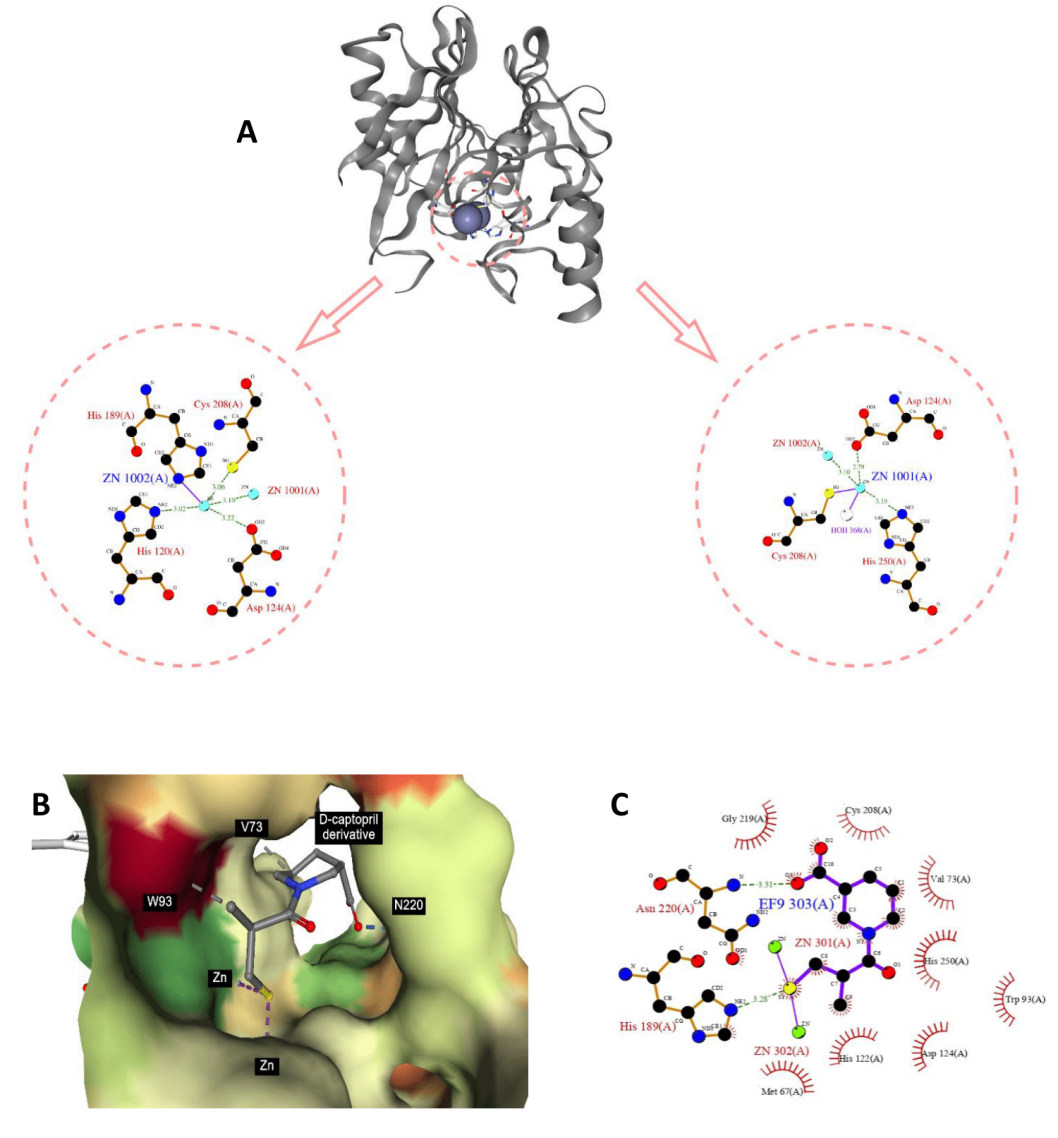
NDM-1 crystal structure and active site. From PDB 3S0Z (A). The binding modality of D-captopril derivative against the active site of NDM-1 (PDB ID: 6LJ4) (B) and 2D interaction diagram generated by Ligplot+ (C), which highlight the role of nitrogen atom and the adjacent aliphatic carbons in stabilization of the protein-ligand complex.

From amongst the top 20 ranked features, nitrogen-containing features account for 7 (35 %). They are KRFPC 4080, 3882, 677, 3608, 3750, 4287 and 3943. Amongst the features, KRFPC 677, 3750, 4287 and 3943 belong to amines. Amine is defined as any class of basic organic compounds derived from ammonia by the replacement of hydrogen with alkyl or aryl groups. Amines are nucleophiles that can bond to a variety of electrophiles. Due to the lone pair of electrons, amines are basic in nature, and they can form hydrogen bonds. Shown in Figure 7(Fig. 7) is the ligand-enzyme interaction profile for nitrogen-containing feature. 

The nitrogen has a lone pair of electrons, with 3 substituents, it may bind to a fourth substituent, leaving a positive charge on the nitrogen atom, which can serve as intermediates for important reactions. Amongst NDM-1 inhibitors, there are a number of significant molecules that contain amines, for example, Aspergillomarasmine A (AMA) and derivatives, methisazone, and derivatives, cefaclor, aryl 2-aminoimidazole derivatives (Linciano et al., 2019[46]). Another feature KRFPC 3608, which is the 2-methylpyridine, is widely seen in novel categories of anti-NDM 1 agents. For example, N-acylhydrazones and diaryl-substituted thiosemicarbazones contain 2-methylpyridine groups (Gao et al., 2021[21], Li et al., 2021[44]). Pyridine is a heterocycle with nitrogen. Due to the nitrogen in the ring, which results in relatively lower electron density of the carbon atoms of the ring, pyridine-containing molecules undergo nucleophilic substitution reactions more easily than corresponding benzene derivatives. The feature is present in a few zinc chelators, such as tris (2-pyridylmethyl)amine (TPA) and N,N,N',N'-tetrakis (2-pyridylmethyl)ethylenediamine (TPEN) (Schnaars et al., 2018[67]; He et al., 2020[27]). The tris(2-pyridylmethyl) amine (TPA) is a zinc chelator and its derivatives are synthesized as NDM-1 inhibitors by stripping the zinc ions in the active site. This is a tripodal ligand scaffold that is widely used in the coordination and chelation of zinc ions (Huang et al., 2013[32]). Within the trigonal ligand scaffold, there are three identical 2-methylpyridine groups as the metal coordinator. The 2-methylpyridine groups are important in forming the coordination complex.

#### Sulfur-containing features

**Figure 8:**
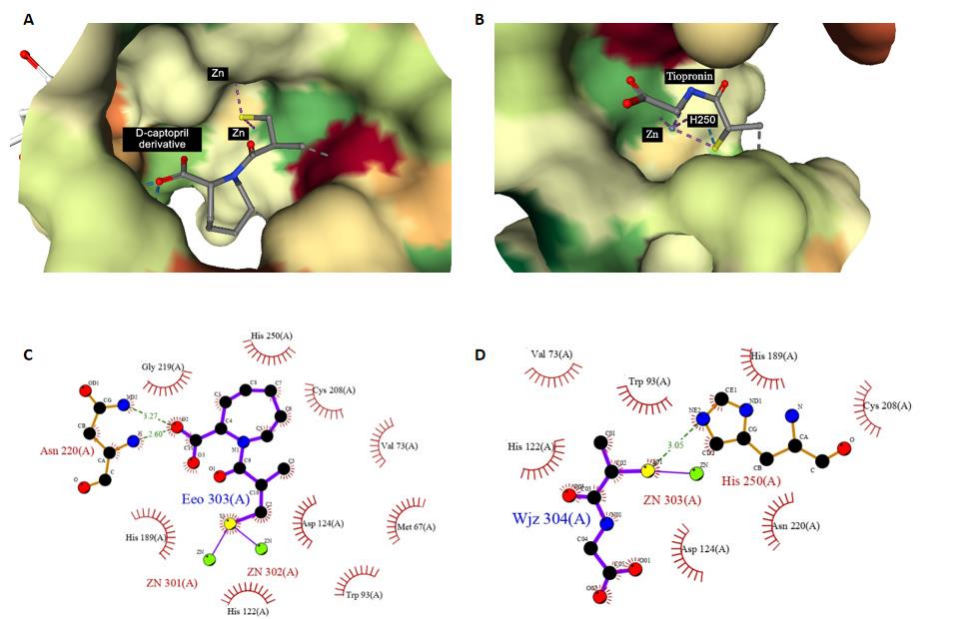
Ligplot illustration of thiol group in ligand/NDM-1 interaction. A and C are the D-captopril derivative from PDB 6LIZ, B and D are the tiopronin from PDB 5A5Z.

There are 2 sulfur-containing features: KRFPC 2855 and KRFPC 4843. KRFPC 2855 is the sulfur with two R groups, while KRFPC 4843 is the indication of sulfur in the molecule. The presence of sulfur in an organic molecule is indicative of an organosulfur compound. Amongst NDM-1 inhibitors, there are many of them containing the sulfur, such as L-captopril, D-captopril, thiol derivatives, mercapto acid derivatives, thioester derivatives, etc (Li et al., 2014[45]; Zhang et al., 2019[95]; Ma et al., 2021[53]). Thiol group is capable of interacting directly with the zinc ion to cripple the enzyme. While for sulfur with two R groups, sulfide group, it can form hydrogen bonds with residues to stabilize the complex as shown in Figure 8(Fig. 8). For example, molecular docking has indicated that sulfur on the amino acid thioester derivative 1 can form hydrogen bond with serine 223, which facilitates the complex and affinity (Zhang et al., 2019[95]). 

#### Aromatic features

There are 6 features that contain aromatic rings. They are KRFPC 1566, 1564, 1642, 3608, 4287 and 3943. KRFPC 1566, 1564 and 1642 are phenyl groups of various substitutions. The presence of phenyl groups amongst NDM-1 inhibitors is very common. For example, ebselen, some thiol-containing compounds, cyclic boric acid derivatives, and pyridinedicarboxylic acid derivatives (Li et al., 2014[45]). The presence of benzene rings is very important for forming hydrophobic interactions and electrostatic interactions with NDM-1 active site residues. For example, in rhodanine derivatives, the benzene rings can form hydrophobic interactions with Gln60, Trp87, Gly160, these interactions facilitate the binding to the active sites to make it more potently anchored (Xiang et al., 2018[87]). On the other hand, benzene rings can affect the water solubility of compounds. The presence of benzene rings can increase the hydrophobicity of molecules. The latter three aromatic features all contain nitrogens, and have been discussed in nitrogen-containing features.

#### Carbonyl features

**Figure 9:**
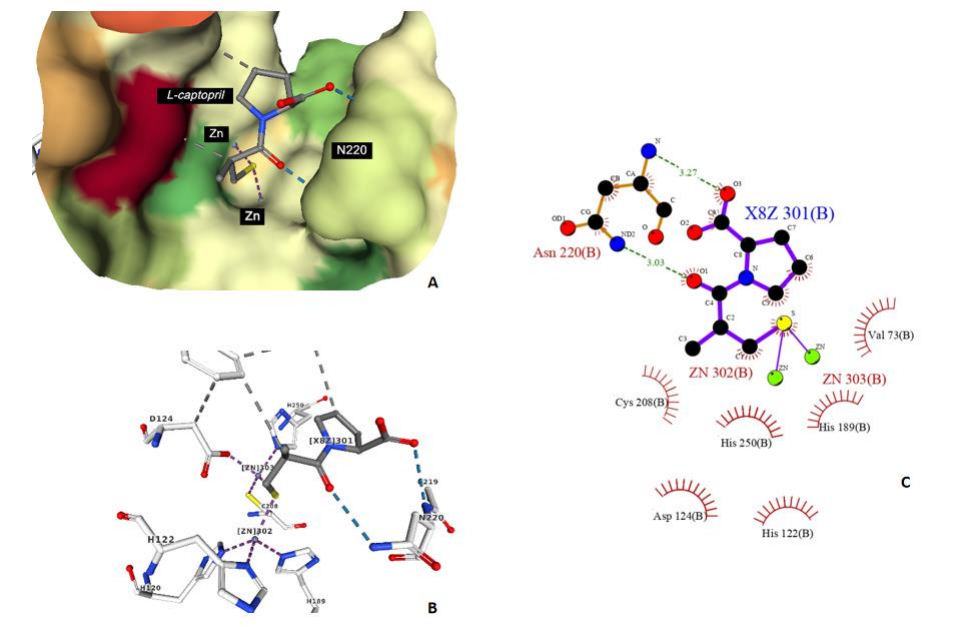
The binding modality of L-captopril against the active site of NDM-1 (PDB ID: 4EXS) (A and B) and 2D interaction diagram generated by Ligplot+ (C), the carbonyl groups act as hydrogen bond acceptor with Asn220 to facilitate the pocket formation.

There are two carbonyl features, KRFPC 1193 and KRFPC 3025. Due to the high polarity of the carbonyl bond, they are polar and hydrogen bond acceptors. Carbonyl groups exist in ketones, aldehydes or carboxylic acids. The carbon-oxygen double bond forms hydrogen bond with adjacent NDM-1 active site residues as shown in Figure 9(Fig. 9).

#### Aliphatic features

**Figure 10:**
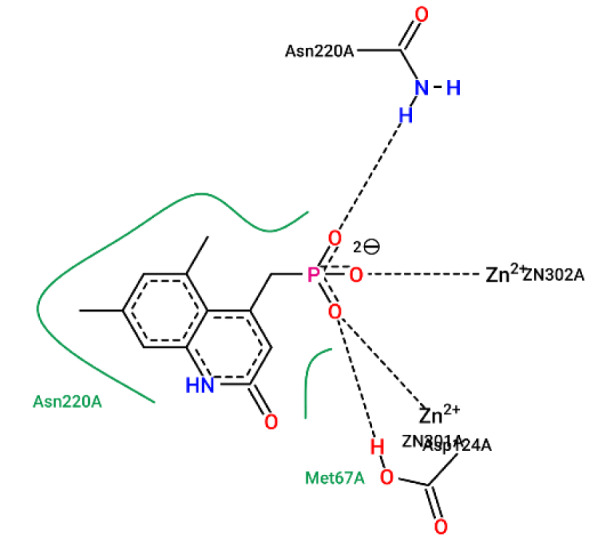
Ligplot illustration of methyl group in ligand/NDM-1 interaction. This is heteroaryl phosphonate derivative with NDM-1, from PDB 6D1J. The two methyl groups form hydrophobicity with Asn220A, marked by the green spline section.

KRFPC 2975, 297, 3224, 669 belong to aliphatic features. KRFPC 669 is the methyl group. As a non-polar functional group, it is important in forming non-polar covalent bonds, or hydrophobic interactions with adjacent residues as shown in Figure 10(Fig. 10).

### Limitation of the study

First and foremost, as this is a pure computational study on drug discovery, there's a lack of *in vitro* experiment support. Secondly, although the study utilizes data from reliable literature and accredited chemical databases, the scale and diversity of the dataset are not large and broad enough to cover more portions of chemical space. Last but not least, currently, there are some novel categories of compounds, such as peptidomimetics, oligomer nucleic acid drugs, nanoparticles (Sully et al., 2017[76]; Kazi et al., 2020[38]), etc that may play roles as NDM-1 inhibitors, this study doesn't involve these novel compounds. This is also due to the lack of related data in this field.

## Conclusion

Antimicrobial resistance is a significant global challenge. NDM-1 as a metallo-beta-lactamase is one of the most perplexing factors in public health and infection control by causing antimicrobial resistance. However, until now, there are no effective drugs for NDM-1 and the structure-activity relationship remains largely unknown. In this study, a QSAR model using currently available NDM-1 inhibiting compounds has been made, via a random forest algorithm and 12 sets of molecular fingerprints from the PaDEL package. And metrics for model performance evaluation have indicated that KlekotaRothCount fingerprint set provides the best performance and model robustness. Feature importance ranking after hyperparameter tuning has demonstrated that amine group, 2-methylpyridine group, sulfur, aromatic features, carbonyl groups and certain aliphatic hydrocarbons are the features that contribute to NDM-1 inhibiting activities. The findings from this study can facilitate drug discovery of NDM-1 inhibitors and can be a guideline for further optimization. 

## Notes

Chanin Nantasenamat and Theeraphon Piacham (Department of Clinical Microbiology and Applied Technology, Faculty of Medical Technology, Mahidol University, Bangkok 10700, Thailand; E-mail: theeraphon.pia@mahidol.ac.th) contributed equally as corresponding author.

## Declaration

### Acknowledgments

This research project is supported from National Research Council of Thailand (NRCT) and Mahidol University: NRCT5-RSA63015-17, the Center of Excellence on Medical Biotechnology (CEMB), S&T Postgraduate Education and Research Development Office (PERDO), Office of Higher Education Commission (OHEC), Thailand.

### Conflict of interest

The authors declare no conflict of interest.

## Supplementary Material

Supplementary information

## Figures and Tables

**Table 1:**
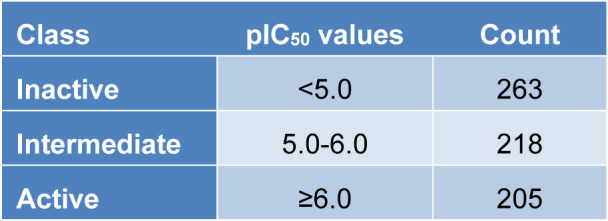
Summary of counts of NDM-1 inhibitors along with bioactivity information. After data balancing, all three classes have the same size of 263.

**Table 2:**
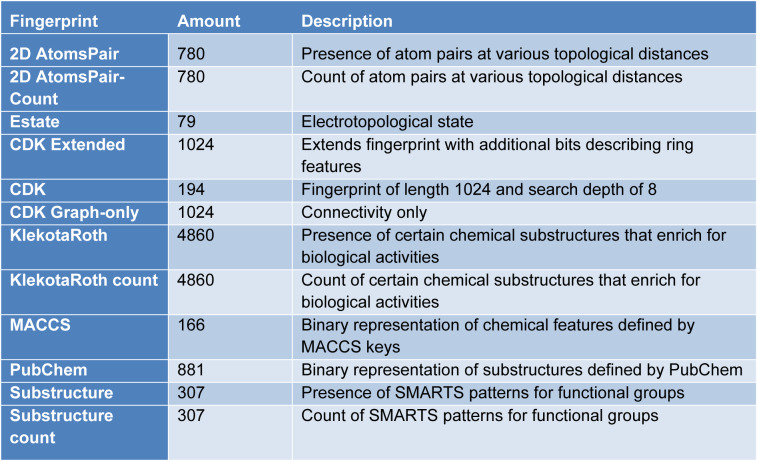
All 12 sets of molecular fingerprints in PaDEL software

**Table 3:**
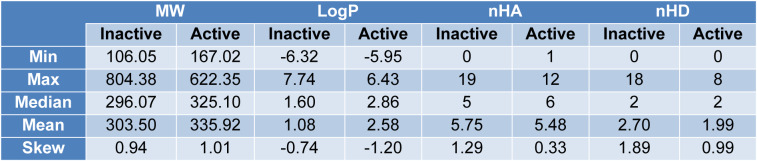
Lipinski descriptor statistics of inactive and active classes

**Table 4:**
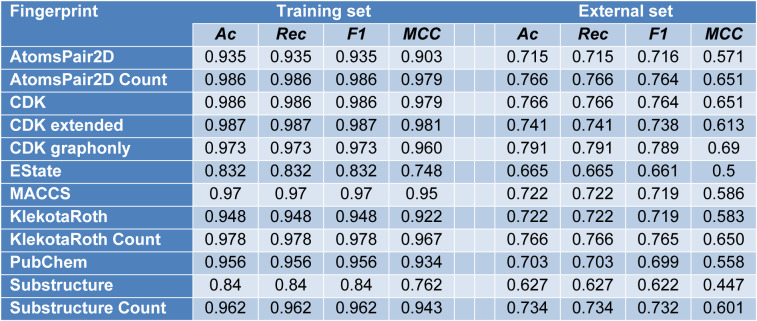
Summary of model performance built using 12 sets of molecular fingerprints using default model hyperparameters (n_estimators = 500, max_features = 3, random state=42)

**Table 5:**
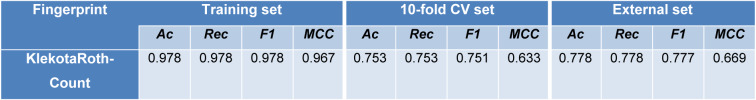
Summary of performance for random forest model built with tuned hyperparameters (n_estimators =150, max_features=5, random state=42).

**Table 6:**
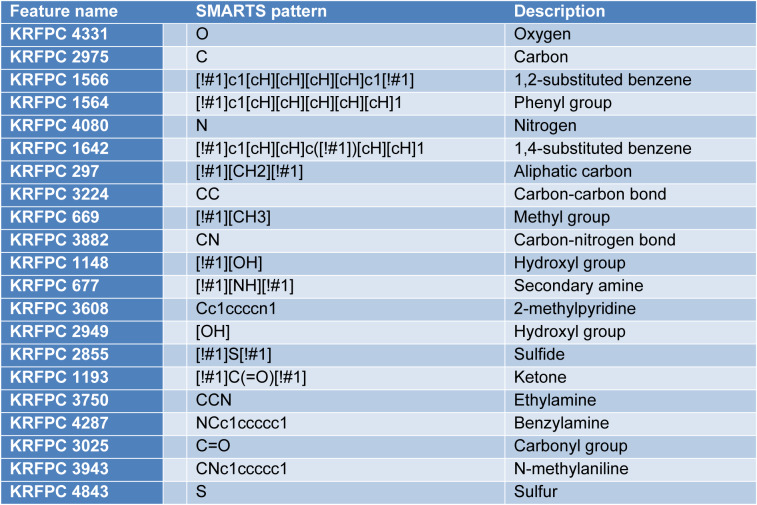
Feature importance from RF model built using KlekotaRothCount fingerprint
