# Supplementary material for: Towards combating antibiotic resistance by exploring the quantitative structure-activity relationship of NDM-1 inhibitors
Source: EXCLI J. 2022 Nov 16;21:1331–51. doi: 10.17179/excli2022-5380 (PMC9755517; doi:10.17179/excli2022-5380)
Supplement: Supplementary information [file EXCLI-21-1331-s-001.pdf]

EXCLI Journal 2022;21:1331-1351 – ISSN 1611-2156
Received: August 30, 2022, accepted: November 10, 2022, published: November 16, 2022

Supplementary information to:

Original article:

TOWARDS COMBATING ANTIBIOTIC RESISTANCE BY EXPLORING THE QUANTITATIVE STRUCTURE-ACTIVITY RELATIONSHIP OF NDM-1 INHIBITORS

Tianshi Yu[1,2], Aijaz Ahmad Malik[3], Nuttapat Anuwongcharoen[1], Warawan Eiamphungporn[2], Chanin Nantasenamat[4*], Theeraphon Piacham[2*]

[1] Center of Data Mining and Biomedical Informatics, Faculty of Medical Technology, Mahidol University, Bangkok 10700, Thailand
[2] Department of Clinical Microbiology and Applied Technology, Faculty of Medical Technology, Mahidol University, Bangkok 10700, Thailand
[3] Center of Excellence in Computational Molecular Biology, Faculty of Medicine, Chulalongkorn University, Bangkok, Thailand
[4] Streamlit Open Source, Snowflake Inc., USA

* **Corresponding authors:** Chanin Nantasenamat, Streamlit Open Source, Snowflake Inc., USA. E-Mail: hellodataprofessor@gmail.com
Theeraphon Piacham, Department of Clinical Microbiology and Applied Technology, Faculty of Medical Technology, Mahidol University, Bangkok 10700, Thailand.
E-mail: theeraphon.pia@mahidol.ac.th

https://dx.doi.org/10.17179/excli2022-5380

This is an Open Access article distributed under the terms of the Creative Commons Attribution License (http://creativecommons.org/licenses/by/4.0/).

Supplementary Table 1: Sources of significant scaffolds of NDM-1 inhibitors from primary literature. The representative molecules and scaffolds are not the whole dataset, instead, they are the significant scaffolds, as well as several representative natural products.

No.	Molecules	Scaffold	Count	Year	Reference
1	Aspergillomarasmine A derivatives	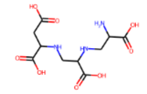	10	2017	Zhang et al., 2017
2	Captopril analogues	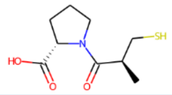	21	2014	Li et al., 2014
3	Thiol-based inhibitor	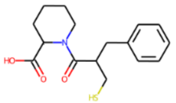	15	2018	Büttner et al., 2018
4	Amino acid thioester derivative	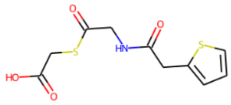	5	2015	Liu et al., 2015
5	((S)-3-Mercapto-2-methylpropanamido)acetic acid derivatives	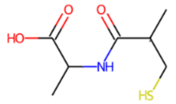	12	2018	Liu et al., 2018a
6	Thiol-based inhibitor	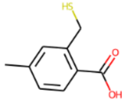	14	2018	Cain et al., 2018
7	Mercaptocarboxylic acids with bioisoteric groups	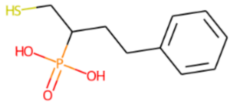	11	2017	Skagseth et al., 2017
8	Isatin-β-thiosemicarbazones	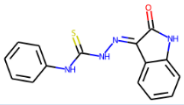	14	2018	Song et al., 2018
9	Triazolylthioacetamide	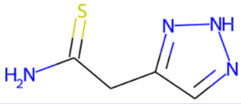	24	2016	Zhai et al., 2016
10	Azolylthioacetamides	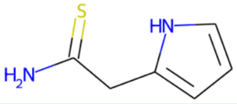	9	2017	Xiang et al., 2017
11	Rhodanine derivatives	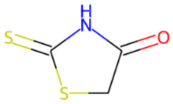	17	2018	Xiang et al., 2018
12	Dipicolinic acid derivatives	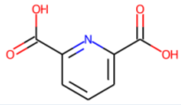	29	2018	Chen et al., 2017

EXCLI Journal 2022;21:1331-1351 – ISSN 1611-2156
Received: August 30, 2022, accepted: November 10, 2022, published: November 16, 2022

No.	Molecules	Scaffold	Count	Year	Reference
13	6-phosphonomethylpyridine-2-carboxylates	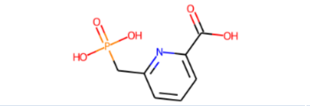	3	2018	Hinchliffe et al., 2018
14	Cyclic boronates	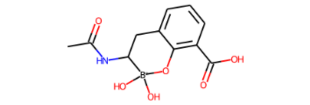	5	2016	Brem et al., 2016b
15	Boronic acids	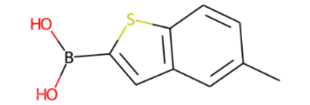	6	2017	Santucci et al., 2017
16	Chromone scaffold	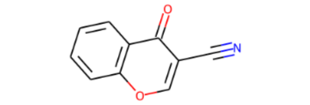	1	2016	Christopeit and Leiros, 2016
17	Thiophene-carboxylic acid derivatives	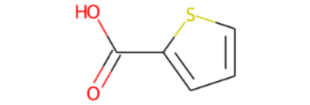	3	2013	Shen et al., 2013
18	Rosmarinic acid and salvianolic acid A	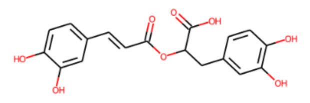 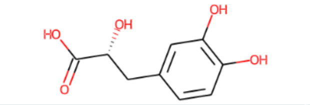	3	2018	Yu et al., 2018
19	Captopril analogues	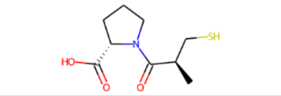	5	2016	Brem et al., 2016a
20	Magnolol, natural product	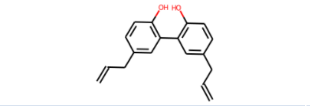	1	2018	Liu et al., 2018b
21	TPA, metal chelator	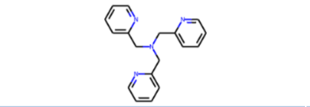	1	2018	Schnaars et al., 2018
22	*N*-Sulfonyl Pyrrole-2-carboxylates	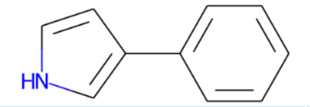	9	2021	Farley et al., 2021
23	Thiosemicarbazone derivatives	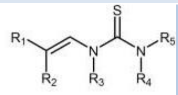	2	2021	Zhao et al., 2021

EXCLI Journal 2022;21:1331-1351 – ISSN 1611-2156
Received: August 30, 2022, accepted: November 10, 2022, published: November 16, 2022

No.	Molecules	Scaffold	Count	Year	Reference
24	Benzimidazole and benzox-azole zinc chelators	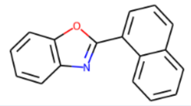	6	2021	Jackson et al., 2021
25	D-captopril derivatives	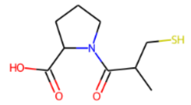	10	2021	Ma et al., 2021
26	Carnosic acid, natural product	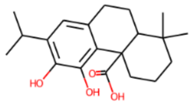	1	2020	Yang et al., 2020
27	Triazole inhibitors	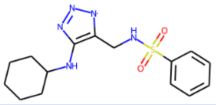	6	2020	Muhammad et al., 2020
28	Oleanolic acid analogues	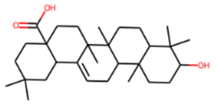	1	2020	Zhou et al., 2020
29	H2 DEDPA derivatives	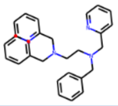	16	2020	Cui et al., 2020
30	Cephalosporin prochelator	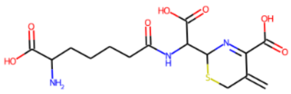	1	2020	Jackson et al., 2020
31	Iminodiacetic acid derivatives	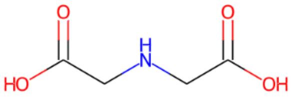	12	2020	Chen et al., 2020
32	Ebselen derivative	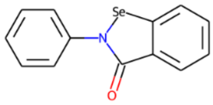	1	2020	Jin et al., 2020
33	Small molecule carboxylates	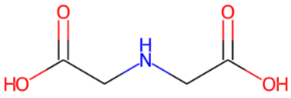	5	2020	Tehrani et al., 2020a
34	Isoliquiritin, natural product	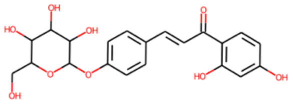	1	2020	Wang et al., 2020
35	Aminocarboxylic acid	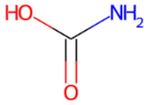	15	2020	Tehrani et al., 2020b

No.	Molecules	Scaffold	Count	Year	Reference
**36**	3-Bromopyruvate	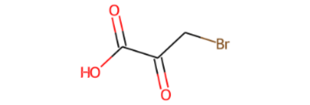	1	2020	Kang et al., 2020
**37**	Selenium-containing scaffold	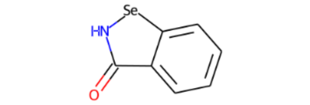	20	2019	Chen et al., 2019
**38**	Dithiocarbamate scaffold	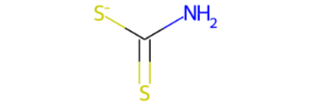	10	2019	Ge et al., 2019
**39**	Pterostilbene, natural product	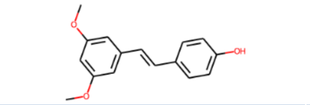	1	2019	Liu et al., 2019a
**40**	Baicalin, natural product	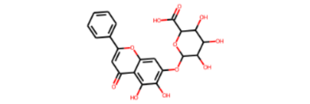	1	2019	Shi et al., 2019
**41**	ZINC84525623	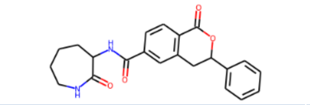	1	2019	Rehman et al., 2019
**42**	Azolylthioacetamide derivatives	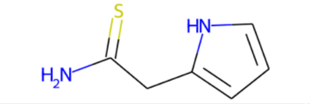	11	2019	Liu et al., 2019b
**43**	Ebsulfur scaffold	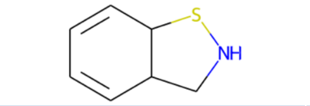	19	2019	Su et al., 2019
**44**	Amino acid thioesters	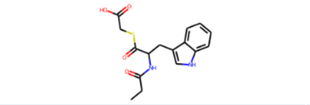 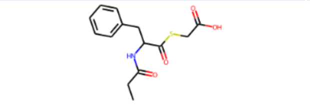 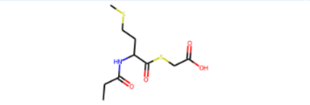	7	2019	Zhang et al., 2019
**45**	Phosphonamidate monoesters	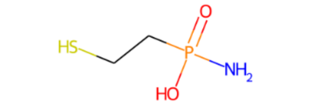	2	2022	Palica et al., 2022

EXCLI Journal 2022;21:1331-1351 – ISSN 1611-2156
Received: August 30, 2022, accepted: November 10, 2022, published: November 16, 2022

No.	Molecules	Scaffold	Count	Year	Reference
46	Fisetin, natural product	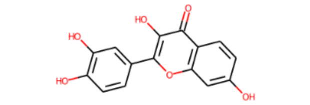	1	2022	Guo et al., 2022
47	Emerione A, natural product	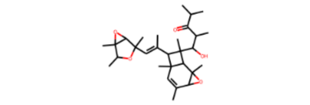	2	2022	He et al., 2022
48	*N*-aryl mercaptoacetamide derivatives	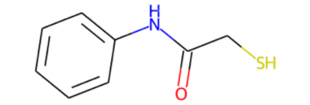	13	2021	Yahiaoui et al., 2021
49	1,2,4-Triazole-3-thione	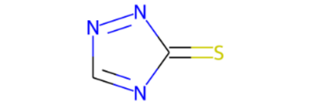	1	2021	Legru et al., 2021
50	H2 DEDPA derivatives	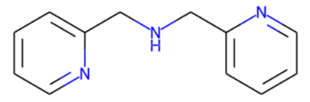	5	2021	Chen et al., 2021
51	*N*-acylhydrazone derivatives	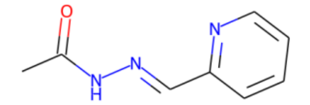	11	2021	Gao et al., 2021
52	Ethane-1,2-dithiol and propane-1,2-dithiol	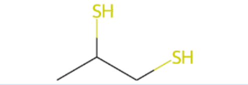 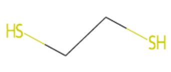	24	2021	Krasavin et al., 2021
53	Aspergillomarasmine A derivatives	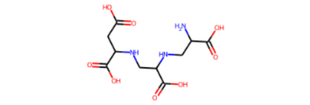	23	2022	Koteva et al., 2022
54	Unithiol drug repositioning	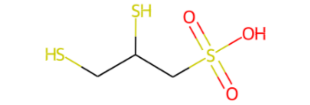	1	2022	Grigorenko et al., 2022
55	Thiosemicarbazones	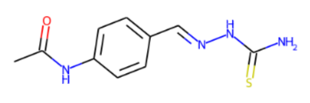	13	2021	Ge et al., 2021
56	8-hydroxyquinoline-7-car-boxylic acid derivatives	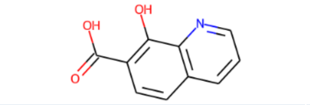	15	2021	Shin et al., 2021
57	Hydroxamate derivatives	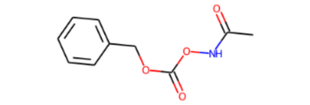	13	2022	Chigan et al., 2022b

EXCLI Journal 2022;21:1331-1351 – ISSN 1611-2156
Received: August 30, 2022, accepted: November 10, 2022, published: November 16, 2022

No.	Molecules	Scaffold	Count	Year	Reference
**58**	Quinolinyl sulfonamides and sulphonyl esters	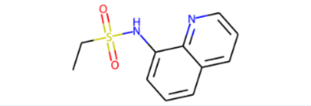 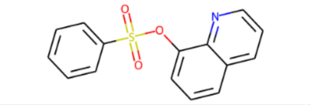	26	2022	Chigan et al., 2022a
**59**	Cephalosporin analogues	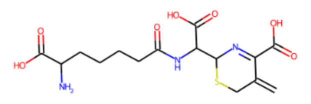	24	2022	Hu et al., 2022
**60**	Cephalosporin prodrugs	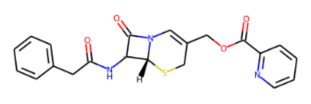	6	2021	van Haren et al., 2021
**61**	Dipyridyl-substituted thio-semicarbazone	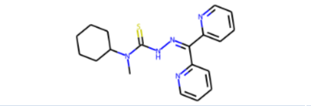	2	2021	Li et al., 2021a
**62**	Diaryl-substituted thiosemicarbazone	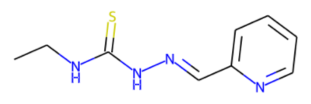 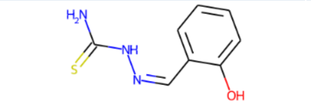 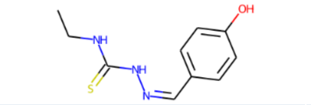	26	2021	Li et al., 2021b
